# Emerging concepts in the diagnosis and treatment of patients with undifferentiated angioedema

**DOI:** 10.1186/1865-1380-5-39

**Published:** 2012-11-06

**Authors:** Jonathan A Bernstein, Joseph Moellman

**Affiliations:** 1 Department of Internal Medicine, Division of Immunology/Allergy, University of Cincinnati Medical Center, 231 Albert Sabin Way, PO Box 670563, Cincinnati, OH, 45267-0550, USA; 2 Emergency Medicine, University of Cincinnati Medical Center, Cincinnati, OH, USA

## Abstract

Angioedema is a sudden, transient swelling of well-demarcated areas of the dermis, subcutaneous tissue, mucosa, and submucosal tissues that can occur with or without urticaria. Up to 25% of people in the US will experience an episode of urticaria or angioedema during their lifetime, and many will present to the emergency department with an acute attack. Most cases of angioedema are attributable to the vasoactive mediators histamine and bradykinin. Histamine-mediated (allergic) angioedema occurs through a type I hypersensitivity reaction, whereas bradykinin-mediated (non-allergic) angioedema is iatrogenic or hereditary in origin.

Although their clinical presentations bear similarities, the treatment algorithm for histamine-mediated angioedema differs significantly from that for bradykinin-mediated angioedema. Corticosteroids, and epinephrine are effective in the management of histamine-mediated angioedema but are ineffective in the management of bradykinin-mediated angioedema. Recent advancements in the understanding of angioedema have yielded pharmacologic treatment options for hereditary angioedema, a rare hereditary form of bradykinin-mediated angioedema. These novel therapies include a kallikrein inhibitor (ecallantide) and a bradykinin β2 receptor antagonist (icatibant). The physician’s ability to distinguish between these types of angioedema is critical in optimizing outcomes in the acute care setting with appropriate treatment. This article reviews the pathophysiologic mechanisms, clinical presentations, and diagnostic laboratory evaluation of angioedema, along with acute management strategies for attacks.

## Review

Up to 25% of people in the US will experience an episode of urticaria, angioedema, or both at some point during their lifetime. It is estimated that each year more than 1 million patients present to a physician with signs or symptoms of urticaria or angioedema, many of whom present to the emergency department with an acute attack [1-3]. Symptoms of urticaria are similar to those of allergic angioedema and may be a component of anaphylaxis [1,4].

Although both urticaria and allergic angioedema are mediated by the activation of mast cells, there are many differences between the two conditions. Unlike angioedema, urticaria rarely affects mucosal tissue. Urticarial wheals involve both the mid- and papillary dermis, whereas angioedema involves the reticular (deep) dermis and subcutaneous and submucosal tissues. Isolated angioedema can sometimes manifest with symptoms of pain and tenderness, whereas itching can be present with or without urticaria in patients with angioedema [3,5].

Angioedema is a presenting sign that results from an underlying pathophysiologic process involving the localized or systemic release of one of several vasoactive mediators, most frequently histamine or bradykinin. Angioedema resulting from the biochemical cascade initiated by the release of bradykinin is distinct from that caused by histamine release; however, the resulting clinical signs and symptoms may be quite similar. Both mediators induce vascular leakage and consequent non-pitting interstitial edema, which results in transient swelling of well-demarcated areas. Although angioedema may occur at any site of the body, it most commonly involves the head, neck, lips, mouth, tongue, larynx, and pharynx, along with the subglottal, abdominal, and genital areas [1,3,6,7].

Angioedema can progress rapidly, and cases that involve the mouth, tongue, larynx, lips, or face constitute a medical emergency. Swelling of these tissues can occur in a matter of minutes in the case of histamine-mediated angioedema compared with a typical slower onset with bradykinin-mediated angioedema. However, both forms of angioedema can lead to imminent airway obstruction and a life-threatening emergency. Thus, emergency physicians must have a basic understanding of the pathophysiologic processes involved in acute angioedema. This review focuses on angioedema induced by histamine or bradykinin release, and not pseudoallergic and idiopathic angioedema, which are discussed only briefly [1].

### Forms of angioedema

Histamine-mediated angioedema occurs through an allergic mechanism, specifically a type I hypersensitivity reaction, which occurs after a patient has had prior “sensitization” to a particular antigen. Upon re-exposure to that antigen, mast cells are activated and release preformed mediators such as histamine and newly formed mediators such as leukotrienes. Increased concentrations of histamine and these other bioactive mediators are responsible for the characteristic edema and swelling that occur during an acute attack.

In general, non–histamine-mediated angioedema occurs through the increased production of bradykinin due to a lack of regulation of the contact pathway, ultimately leading to edema. Bradykinin-mediated angioedema is divided into three distinct types: hereditary angioedema (HAE), angiotensin-converting enzyme inhibitor (ACEI)-induced angioedema, and acquired angioedema (AAE) [1].

Similarities between the clinical presentations of different types of angioedema complicate their management. Although diagnostic blood tests can be very helpful in differentiating between the different types of angioedema instigating an acute attack, performing these tests takes time and results usually cannot be obtained immediately during the acute emergency treatment of an attack. In such cases, achieving a positive clinical outcome depends heavily on the clinician’s ability to distinguish among the different types of angioedema at the bedside through a comprehensive history and physical examination [8].

Importantly, other forms of angioedema exist that are relatively rare, do not occur through an allergic mechanism, and are provoked by the release of a vasoactive mediator other than histamine or bradykinin. These other forms include pseudoallergic angioedema (PAE) and idiopathic angioedema (IAE) [1].

PAE is a form of drug-induced, non-allergic angioedema, and its pathogenesis is related to the mechanism of action of the inciting medication. One example of PAE is the allergic reaction to aspirin and nonsteroidal anti-inflammatory drugs (NSAIDs), where severe bronchoconstriction, severe laryngeal angioedema, urticaria, or shock occurs within 3 to 4 h of ingestion of the drug. PAE in response to aspirin is thought to occur through the inhibition of cyclooxygenase and consequent generation of cysteinyl leukotrienes, which serve as mediators for the resultant angioedematous reaction [1,9].

IAE, which is not well understood, is a diagnosis of exclusion assigned to cases of recurrent angioedema for which no exogenous agent or underlying genetic abnormality can be identified. Some authors have included urticaria-associated angioedema in this category, while others have restricted the diagnosis of IAE to patients with recurrent angioedema without urticaria [10].

### Pathophysiology of angioedema

In general, the pathophysiology of angioedema involves a sudden increase in the permeability of vessel walls in the skin and submucosa. This increased permeability permits local extravasation of plasma and consequent tissue swelling [5].

#### Histamine-mediated angioedema

**Figure 1:**
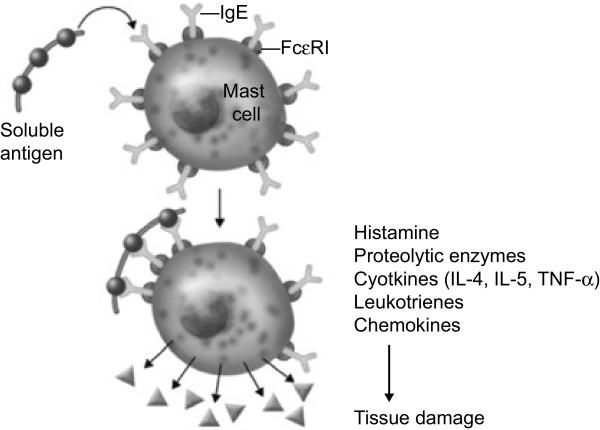
**Type I hypersensitivity is mediated by IgE and induces mast cell degranulation.** FcεRI, high-affinity IgE receptor; IgE, immunoglobulin E; IL-4, interleukin 4; IL-5, interleukin 5; TNF-α, tumor necrosis factor-alpha [4].

Histamine-mediated or allergic angioedema occurs through a type I IgE-mediated hypersensitivity immune response, which is largely mast cell-dependent. Genetically susceptible individuals with prior exposure to an offending allergen become “sensitized.” Sensitization occurs when the allergen is taken up by antigen-presenting cells (i.e., dendritic cells, macrophages, or B cells) and is broken down into small peptides (9–11 amino acids in length). The relevant peptides are then presented to the cell surface in conjunction with major histocompatibility class 2 (MHC2) antigens. This MCH2 peptide complex is recognized by T-helper lymphocyte receptors and a number of other co-stimulatory molecules, resulting in T-cell activation and the release of Th2 cytokines, including interleukin (IL)-4, IL-5, and IL-13, that promote increased production of IgE and the differentiation and migration of eosinophils, in addition to many other functions leading to allergic inflammation. These cytokines also cause B lymphocytes to differentiate into plasma cells that produce specific IgE antibodies that specially recognize the original sensitizing antigenic peptide. These specific antibodies bind to high-affinity IgE receptors (FcεR-1) and can persist on these receptors for months or years. Upon re-exposure to the inciting agent, the allergenic peptide is recognized by the antigen-binding sites of the specific IgE antibodies bound to the high-affinity IgE receptors, leading to a series of chemical reactions that result in activation of the mast cell and the release of preformed and newly formed bioactive mediators (Figure 1) [4]. These mediators, such as histamine, can then bind to selective receptors (i.e., H_1_ receptors) on the vascular endothelium, leading to vasodilation and increased permeability [4,11].

#### Bradykinin-mediated angioedema

**Figure 2:**
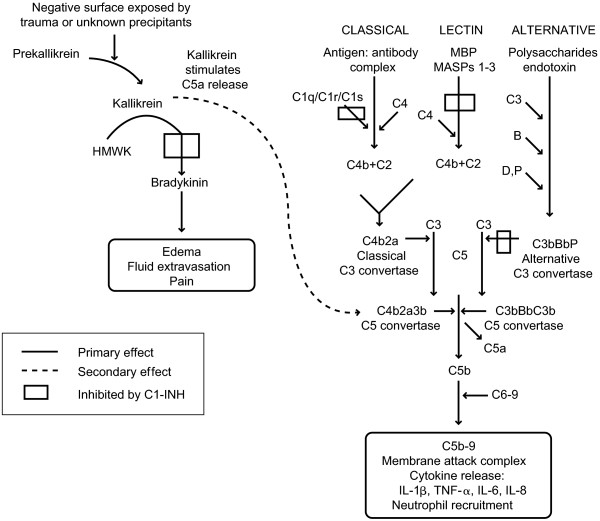
**Kallikrein-kinin cascade.** During an acute HAE attack, reduced activity of C1 esterase inhibitor (C1-INH) results in overactivation of the kallikrein-kinin cascade and subsequent production of bradykinin. Bradykinin is the likely mediator of the vasodilation, edema, and pain that characterize acute HAE attacks. HMWK, high-molecular-weight kininogen; IL, interleukin; MASP, MBP-associated serine protease; MBP, mannose-binding protein; TNF-α, tumor necrosis factor-alpha.

Kinins are a group of pharmacologically active peptides that are released into body fluids and tissues following the enzymatic action of kallikreins on kininogens, which occurs through a complex proteolytic cascade of events called the kallikrein-kinin cascade (Figure 2). The kallikrein-kinin cascade, also referred to as the “contact activation pathway” or intrinsic pathway, is initiated when factor XII (Hageman factor) binds to damaged tissue, becoming activated through conversion to factor XIIa. Factor XIIa converts prekallikrein to plasma kallikrein, and these two proteins autoactivate each other through a positive feedback loop. Plasma kallikrein then cleaves high-molecular-weight kininogen (HMWK), thereby liberating bradykinin [12]. The binding of bradykinin to bradykinin β2 receptors induces vasodilation and increased endothelial permeability, yielding the characteristic signs and symptoms of an acute attack of angioedema [1,13].

##### Hereditary angioedema

HAE is a rare (1:10,000-1:50,000 prevalence), autosomal dominant disorder characterized by a quantitative (type I) or qualitative (type II) deficiency of C1 esterase inhibitor (C1-INH) due to a mutation of the C1-INH *SERPING1* gene, located on chromosome 11q. HAE with normal C1-INH (type III) occurs because of one of two known mutations in the gene for factor XII [10,13,14]. Because C1-INH is a key inhibitor of three enzymes in the kallikrein-kinin cascade—factor XIIa, factor XIIf, and plasma kallikrein—deficiency of functional C1-INH in patients with HAE results in the uncontrolled activation of the entire cascade [13].

**Figure 3:**
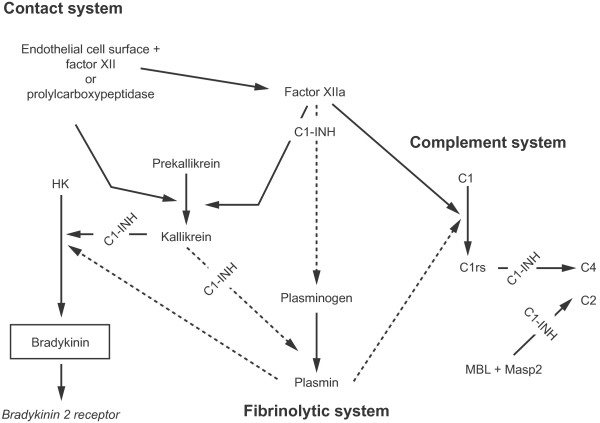
**The contact, complement, and fibrinolytic systems.** C1-INH, C1 esterase inhibitor. Republished with permission from [37]. [PERMISSION PENDING].

In an acute attack of HAE, relative overactivation of the kallikrein-kinin cascade generates excessive bradykinin. Consequently, the vasodilator properties of bradykinin augment vascular permeability, eliciting the characteristic HAE symptoms of localized swelling, inflammation, and pain (Figure 3) [12].

### ACE inhibitor-induced angioedema

Angiotensin-converting enzyme plays a major role in the renin-angiotensin-aldosterone system, through two proteolytic mechanisms: conversion of angiotensin I to angiotensin II and degradation of bradykinin. These two actions make ACE inhibition a chief target in the treatment of hypertension, myocardial infarction (MI), heart failure, and type I diabetic nephropathy. Treatment with an ACEI following MI improves survival, rate of hospitalization, symptoms, and cardiac performance; in addition to the low cost of these agents, these factors account for the widespread use of ACEIs [15,16].

ACEI-induced angioedema is associated with the reduction in bradykinin degradation that is caused by ACEIs. As in HAE, increased levels of bradykinin lead to the symptoms of swelling, pain, and inflammation that are seen in patients who present with an acute attack. ACEI-induced angioedema most often involves the head, neck, face, lips, tongue, and larynx. Rarely, it involves visceral organs. Life-threatening edema of the upper airway presents in 25-39% of cases of ACEI-induced angioedema. Although studies have noted that ACEI-induced angioedema most commonly occurs shortly after treatment is initiated, it can develop long after treatment has started [17]. Interestingly, angiotensin receptor blockers (ARBs), also referred to as AT_1_-receptor antagonists or blockers, appear to induce angioedema at a lower frequency than do ACEIs [1]. When ACE activity is inhibited, the enzyme aminopeptidase P (APP) metabolizes bradykinin. Bradykinin amasses during ACE inhibition in individuals who have subnormal activity of APP due to a genetic mutation in a gene-encoding membrane–bound APP [12].

#### Acquired angioedema

The prevalence of AAE is believed to be 1:100,000 to 1:500,000, and it primarily affects adults and the elderly. AAE results from a non-genetic C1-INH deficiency. Ten to fifteen percent of patients have an underlying lymphoproliferative disorder; therefore, screening these patients with blood tests and possibly bone marrow biopsy to exclude malignancy is recommended. Many of these patients may also have an autoantibody to C1-INH. Treatment of the underlying lymphoproliferative disorder and/or the C1-INH autoantibody can be curative [18].

### Clinical manifestation of angioedema

Patients with angioedema may present with or without urticaria [8]. Angioedematous lesions tend to be non-pitting and non-pruritic. Despite their non-pruritic nature, these lesions can invoke significant sensations of pain and burning [7]. Although they do not in themselves appear desquamated or discolored, the pruritic component of angioedematous lesions may cause scratching or rubbing, with resultant discoloration [6].

#### Histamine-mediated angioedema

Some of the classic signs associated with histamine-mediated angioedema are the “wheal and flare” reaction of the superficial layers of the skin and interstitial edema of underlying subcutaneous, mucosal, and submucosal layers of the skin [4]. These reactions therefore frequently manifest as pruritic hives with or without angioedema. Also of importance is the evanescent nature of these attacks in contrast to non-histamine-mediated angioedema. Acute attacks of urticaria and/or angioedema are typically self-limited; swelling typically lessens or resolves over the course of 24 h. Not infrequently, these reactions can be recurring, and when they persist for more than 6 weeks are considered chronic [5].

Although histamine-mediated attacks of angioedema most commonly occur in hyperallergic or atopic individuals (i.e., patients with allergic rhinitis, extrinsic asthma, or atopic dermatitis/eczema), attacks induced by a food or medication may be seen in the absence of atopy. In addition to acute swelling and edema, allergic angioedema always involves a recognizable trigger, most commonly insect stings, food, or medications [10].

Angioedema that is mediated by histamine typically responds to antihistamines (Table 1). Swelling can occur at any site of the body, but histamine-mediated angioedema has a predilection for the facial area, particularly the lips and periorbital area and, less commonly, the genitalia. Isolated allergic angioedema may involve the throat or larynx, resulting in dyspnea or stridor caused by laryngeal edema. In some instances, patients can progress to anaphylaxis, a potentially fatal systemic allergic reaction [4]. Anaphylaxic manifestations can include diffuse hives, angioedema, gastrointestinal symptoms, and hypotension. In its most severe form, loss of consciousness due to vascular collapse may occur [4]. Pulmonary symptoms, including hyperinflation, peribronchial congestion, submucosal edema, edema-filled alveoli, and eosinophilic infiltration are often noted during anaphylaxis [4]. Although these cases are responsive to antihistamine therapy, identifying a specific cause can be elusive. Often patients and/or physicians implicate a food or drug as the trigger without adequately proving cause and effect, which can lead to erroneous elimination of important medications or unnecessarily restrictive diets. Therefore, once stabilized, these patients should be evaluated by a physician experienced in the management of urticaria/angioedema to establish whether these reactions are secondary to a specific cause or are idiopathic, as the latter is often the case.

**Table 1:** Clinical and diagnostic features of various types of angioedema

**Angioedema type**	**Clinical and diagnostic features**
**Histamine-mediated**	
Allergic angioedema	Angioedema usually accompanied by urticaria and sometimes anaphylaxis; may be pruritic; associated with exposure to allergens; attacks last for 24–48 h; responsive to antihistamines or corticosteroids
Angioedema with urticarial vasculitis	Angioedema accompanied by urticaria; there may be petechiae or purpura after swelling resolves; symptoms of underlying vasculitis
**Bradykinin-mediated**	
Hereditary angioedema types I and II	Recurrent attacks without urticaria; erythema marginatum is a cardinal finding; onset in childhood or young adulthood, worsening at puberty; family history in 75% of patients; attacks unresponsive to antihistamines or corticosteroids
Hereditary angioedema type III	Associated with mutations in factor XII; more common in women; may be estrogen dependent; typical onset after childhood; face, tongue, extremity involvement is more frequent than abdominal; recurrent tongue swelling is cardinal symptom; more disease-free intervals than in HAE types I and II; family history of angioedema; attacks unresponsive to antihistamines or corticosteroids
Acquired angioedema	Attacks similar to HAE; onset in middle age or later; no family history; attacks unresponsive to antihistamines or corticosteroids
ACE inhibitor-induced angioedema	History of ACE inhibitor use; no urticaria; face and tongue most frequent sites; more common in blacks and smokers; patients usually can tolerate ARBs
**Not mediated by histamine or bradykinin**	
Idiopathic angioedema	Angioedema sometimes accompanied by urticaria; swelling may persist for up to 48 h; attacks may occur daily; responsive to antihistamines or corticosteroids
Pseudoallergic angioedema	Urticaria typically present; usually class-specific reaction; thought to be mediated by cysteinyl-leukotrienes; includes NSAID-induced angioedema, which occurs because of cyclooxygenase inhibition and subsequent release of cysteinyl-leukotrienes

*ACE* angiotensin-converting enzyme, *ARB* angiotensin receptor blocker, *NSAID* nonsteroidal anti-inflammatory drug.

##### HAE type III

HAE type III was first used to describe a group of women who presented with angioedema similar to that seen with HAE types I and II but without any complement abnormalities. Patients with HAE type III more commonly experience angioedema in the facial region involving the tongue, and lips; in severe cases, they may develop laryngeal edema. Prodromes such as erythema marginatum have not been commonly observed in HAE type III patients [7].

Patients diagnosed with HAE type III usually manifest symptoms later in life and have a well-defined generational history of angioedema (Table 1). A recent study found the mean age of symptom onset for HAE type III to be 26.8 years (SD ± 14.9 years, range = 1–68 years). Another characteristic of this form of angioedema is that it is frequently exacerbated by estrogen surges during pregnancy or by treatment with oral contraceptives and hormonal replacement therapy [19]. The original description of this variant form of HAE was in a family where a gain-of-function mutation in factor XII was observed [20]. However, subsequent investigations of this mutation in other cases of HAE type III have not yielded similar findings, and as of yet the underlying genetic and mechanistic cause of this condition is unknown.

The clinical course of patients with HAE type III typically differs from HAE types I and II in that they have more disease-free intervals during the course of disease. There is still a great deal of uncertainty regarding how to definitively diagnose and treat this condition.

#### Bradykinin-mediated angioedema, HAE types I and II

The most frequently encountered symptoms in HAE types I and II (HAE due to C1-INH deficiency) are skin edema, abdominal pain, and life-threatening laryngeal edema. Skin swelling occurs most commonly in the extremities and less frequently involves the face and other body sites. Abdominal attacks, which are also very prevalent in these patients, are caused by transient edema of the bowel wall and manifest with significant pain, vomiting, and diarrhea due to partial or complete intestinal obstruction, ascites, and hemoconcentration. In up to one-third of patients, erythema marginatum, which is a serpiginous erythematous non-pruritic rash, can manifest before the onset of an attack in patients with either type I or type II HAE; however, urticaria and pruritus are not typically associated with these two types of HAE (Table 1) [7,10].

A recent analysis of 195 patients with HAE found that 54% experienced an average of more than 12 attacks per year; symptom-free years were rare, representing only 370 (6.5%) of the 5,736 patient-years included in the analysis [19]. Although clinical presentation varies, HAE types I and II often present during childhood (Table 1). Most patients experience progressive worsening of symptoms over several hours; these episodes can be quite protracted, lasting from 2 to 5 days without treatment [20].

#### ACE inhibitor-induced angioedema

ACEI-induced angioedema occurs in 0.1-0.7% of patients treated with these agents. The incidence of ACEI-induced angioedema appears to be highest (25%) during the first month of treatment [21] but can occur from months to years after the initiation of treatment. Less commonly, ACEI-induced angioedema has been associated with medications such as NSAIDs—via inhibition of the COX enzyme pathway leading to changes in prostaglandin synthesis [5,11]—and alteplase [22]. Although rare, angioedema can also be induced by ARBs; for the most part, ARBs are considered safe for use by patients who have a history of ACEI-induced angioedema [21].

ACEI-induced angioedema is not associated with urticaria [8,23] and most commonly involves the tongue, lips, and face [21]. ACEI-induced angioedema appears to be four to five times more common in African-American than in Caucasian individuals [21] owing to genetic polymorphisms in APP, a critical enzyme for metabolizing ACEIs.

#### Acquired angioedema

The presentation of AAE is, broadly speaking, similar to that of HAE types I and II, with recurrent attacks of subcutaneous and/or submucosal swelling without urticaria. As mentioned, AAE is much less common than HAE, affecting approximately one-tenth as many patients. Clinical characteristics that differentiate this form of angioedema from HAE are older age (the typical patient is elderly) and the absence of a family history of angioedema [23].

### Differential diagnosis of angioedema

Angioedema is a clinical sign that may be associated with one of several different clinical conditions. In addition to allergic and non-allergic angioedema, in the differential diagnosis for angioedema the following should be ruled out: facial cellulitis, acute contact dermatitis, photodermatitis, Crohn’s disease (particularly if the lips and mouth are involved), dermatomyositis, facial lymphedema, cellulitis, tumid discoid lupus erythematosus, Ascher syndrome, Melkersson-Rosenthal syndrome, and superior vena cava syndrome [5].

### Laboratory evaluation of angioedema

**Figure 4:**
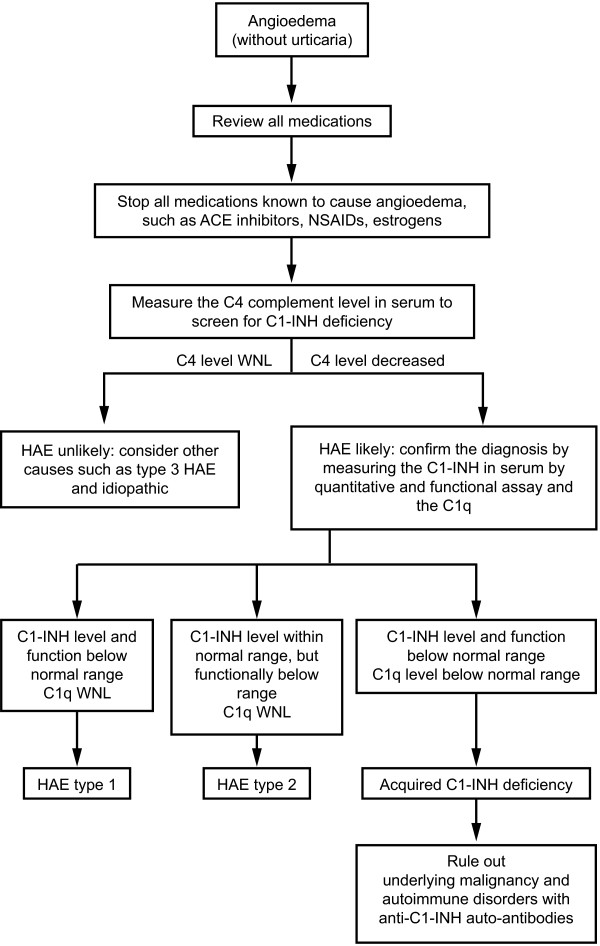
**Diagnostic considerations in patients who present with nonallergic angioedema.** ACE, angiotensin-converting enzyme; NSAIDs, nonsteroidal anti-inflammatory drugs; C1-INH, C1 esterase inhibitor; HAE, hereditary angioedema; WNL, within normal limits. Modified from [8]; with permission. [PERMISSION PENDING].

**Figure 5:**
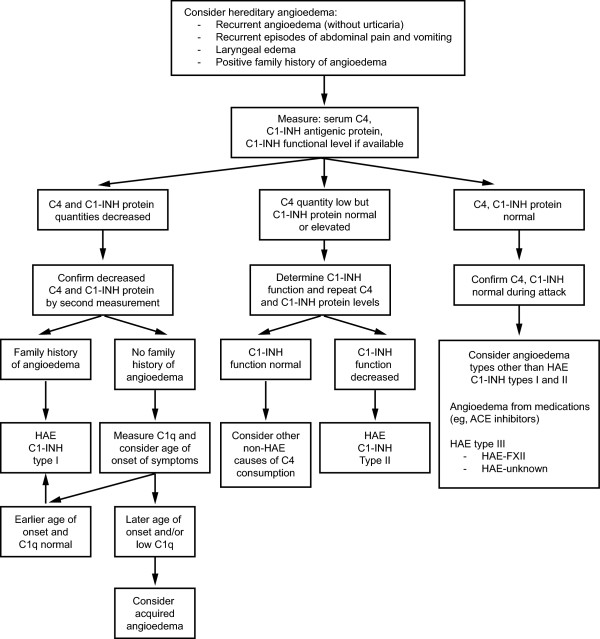
**International consensus algorithm for the diagnosis of hereditary angioedema.** ACE, angiotensin-converting enzyme; C4, complement factor 4; C1-INH, C1 esterase inhibitor; HAE, hereditary angioedema. Modified from [24]; with permission. [PERMISSION PENDING].

A possible algorithm is presented for the diagnostic workup patients with suspected non-allergic angioedema (Figure 4) [8] or HAE (Figure 5) [24].

#### Histamine-mediated angioedema

A definitive diagnosis of histamine-mediated angioedema can be achieved through laboratory evaluation for markers of mast cell degranulation (elevated urine histamine and serum tryptase levels) (Table 2). Prick skin testing or serum-specific IgE assays may be appropriate if the history is suggestive of sensitization to a suspected allergen such as a food. If laboratory test results for urine histamine and serum tryptase levels are unavailable, the diagnosis relies on history and clinical presentation [21].

**Table 2:** Complement profiles involved in each type of angioedema

**Angioedema type**	**Urine histamine**	**Serum tryptase**	**C4 level**	**C1-INH Level (antigenic)**	**C1-INHLevel (functional)**	**C1q level**	**C3 level**
**Histamine-mediated angioedema**	↑	↑	**NL**	**NL**	**NL**	**NL**	**NL**
**Hereditary angioedema types I and II**	**NL**	**NL**	**↓**	**↓ (type I)**	**↓**	**NL**	**NL**
				**NL (type II)**			
**Hereditary angioedema type III**	**NL**	**NL**	**NL**	**NL**	**NL**	**NL**	**NL**
**Acquired angioedema**	**NL**	**NL**	**↓**	**↓ or NL**	**↓**	**↓**	**↓ or NL**
**ACE inhibitor-induced angioedema**	**NL**	**NL**	**NL**	**NL**	**NL**	**NL**	**NL**
**Idiopathic angioedema**	**NL**	**NL**	**NL**	**NL**	**NL**	**NL**	**NL**
**Pseudoallergic angioedema**	**NL**	**NL**	**NL**	**NL**	**NL**	**NL**	**NL**

*ACE* angiotensin-converting enzyme, *C3* complement factor 3, *C4* complement factor 4, *C1-INH* C1 esterase inhibitor, *NL* normal.

#### HAE type III

In patients with HAE Type III, the level and function of C1-INH are normal. The serum C4 level is also normal (Table 2) [8].

#### Bradykinin-mediated angioedema: HAE types I and II

Because the clinical signs and symptoms of HAE types I and II are very similar, distinguishing between the two requires laboratory evaluation. In HAE type I, the serum C4 level is decreased during and between attacks, and the serum C1-INH level is decreased and sometimes undetectable. In HAE type II, the serum C4 level is decreased during and between attacks, while the serum C1-INH level is within normal limits or even increased, but C1-INH is functionally deficient (Table 2, Figures 4 and 5) [8]. Typically, in type II, the serum C2 level is also reduced during attacks, which may be helpful in making the diagnosis [8].

#### ACE inhibitor-induced, idiopathic, and acquired angioedema

Patients who present with attacks of angioedema due to ACEIs will have normal levels of C4 and C1-INH (Table 2, Figure 5) [10]. Similarly, in IAE, C4 levels along with all other laboratory results are normal. IAE is primarily a diagnosis of exclusion (Table 2, Figures 4 and 5), In AAE, C4 levels and complement protein C1q are reduced; C3 levels may be low or normal (Table 2) [1].

### Management of acute attacks of angioedema

**Figure 6:**
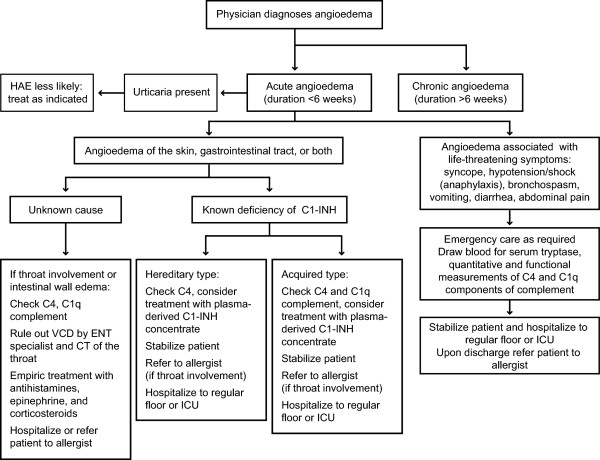
**Algorithm for the management of patients who present with acute angioedema.** C1-INH, C1 esterase inhibitor; CT, computed tomography; ENT, ear, nose, and throat; ICU, intensive care unit; VCD, vocal cord dysfunction. Modified from [8]; with permission [PERMISSION PENDING].

The international consensus from the third international conference on HAE is that for all forms of angioedema, airway patency is the first priority in an acute attack. [24]. An algorithm for the management of acute angioedema (duration < 6 weeks) is presented in Figure 6.

A low threshold for intubation is recommended. Intubation must be performed at the first sign of airway compromise, and all cases involving laryngeal edema are considered a medical emergency [8,14,21].

In cases of angioedema involving the tongue, oral intubation is difficult at best and often impossible. Direct fiberoptic nasotracheal intubation is the preferred method to achieve airway patency in patients with significant laryngeal edema. Blind nasotracheal intubation should be avoided because of the increased potential for localized trauma and consequent worsening of the edema.

During an episode of acute angioedema, it may be technically difficult to insert an endotracheal tube. If time permits, consultation with an otolaryngologist should be obtained for provision of a surgical airway. In the event that all other airway methods have failed and ENT consultation is unavailable, the emergency physician should be prepared to perform a surgical airway [1]. If attempts at nasotracheal intubation are unsuccessful, cricothyrotomy or tracheotomy is indicated [6,14].

In the following sections, acute management approaches for attacks of histamine-mediated angioedema, ACEI-induced angioedema, and HAE, along with drug therapies for HAE, will be addressed. Although a discussion of prophylactic approaches to reduce the risk of subsequent attacks is beyond the scope of this article, such approaches should be considered for all patients following the resolution of an acute attack.

#### Severe histamine-mediated angioedema, or anaphylaxis

The priority of the acute management of angioedema is airway maintenance. Intramuscular (IM) epinephrine may be used to control symptoms and sustain blood pressure during an anaphylactic reaction; it may be life-saving for patients with acute laryngeal edema or anaphylaxis [8]. Epinephrine 1:1,000 is administered IM 0.2-0.5 mg thigh (adults); 0.01 mg/kg (up to 0.03 mg) thigh (children). This dose can be repeated every 5–15 minutes, with close monitoring for signs and symptoms of toxicity [25].

The α-adrenergic, vasoconstrictive effect of epinephrine reverses peripheral vasodilation, which reduces angioedema and urticaria. The β-adrenergic properties of epinephrine cause bronchodilation, increase myocardial output and contractility, and suppress further mediator release from mast cells and basophils. It is important to note that epinephrine, administered in low concentrations (e.g., 0.1 mg/kg) is paradoxically associated with vasodilation, hypotension, and the increased release of inflammatory mediators. Because of the risk for potentially lethal arrhythmias, intravenous (IV) epinephrine 1:10,000 or 1:100,000 dilutions should be administered only during cardiac arrest or to patients who are profoundly hypotensive or have failed to respond to both IV volume replacement and multiple injections of epinephrine [25].

An important component of the acute management of anaphylaxis presented by severe histamine-mediated angioedema is volume expansion. The largest catheter possible should be inserted into the largest peripheral vein, and the rate should be titrated to pulse and blood pressure; infuse 1–2 l normal saline rapidly by IV in adults (5–10 ml/kg in the first 5 min), 30 ml/kg in the first hour in children [25].

Antihistamines act more slowly than epinephrine, have minimal effect on blood pressure, and should not be administered alone as treatment for anaphylaxis or acute allergic angioedema. Combined histamine-receptor blockade, with H_1_ and H_2_ blockers, is more effective than the use of H_1_ agents alone. Diphenhydramine should be administered 25–50 mg IV (adults), 1 mg/kg IV up to 50 mg (children). Identical oral doses may be sufficient for milder episodes. Ranitidine should be administered 1 mg/kg (adults), 12.5-50 mg infused over 10 min (children) [25,26].

Inhaled β2 agonists (e.g., albuterol) are helpful when bronchospasm resists epinephrine injections alone. Systemic corticosteroids are not sufficient to prevent the progression of anaphylaxis [25]. Although the use of a parenteral corticosteroid (IV methylprednisolone) provides a benefit in histamine-mediated angioedema, its therapeutic effect is not immediate.

#### ACE inhibitor-induced angioedema

For all types of angioedema, the priority of acute management is maintenance of airway patency. Because ACEI-induced angioedema does not involve histamine, antihistamines have not been found to be effective in either the acute or long-term management of these patients; similarly, corticosteroids are not effective for these conditions. Epinephrine should be considered to temporarily constrict permeable blood vessels. For patients who present with an acute attack of ACEI-induced angioedema, an immediate first step is discontinuation of the ACEI [6].

The mechanism underlying ACEI-induced angioedema (excess bradykinin) is similar to that underlying HAE. For that reason, agents shown to be effective in HAE, including the plasma kallikrein inhibitor ecallantide and the bradykinin receptor antagonist icatibant, are currently being investigated in clinical studies as treatments for acute ACEI-induced angioedema [27,28]. Several small case studies have reported on the use of icatibant for the treatment of ACEI-induced angioedema [29-31].

#### Hereditary angioedema

In May 2010, the third international conference on HAE was held in Toronto, Canada, where international consensus approaches for the diagnosis, treatment, and management of HAE were reviewed and updated. The consensus documents divide the therapy for patients with HAE into acute treatment, short-term prophylaxis, and long-term prophylaxis. The consensus recommends that HAE attacks be treated as early as possible [24]. Patients with HAE are unlikely to respond to antihistamines or corticosteroids. Epinephrine has low efficacy in HAE, but has been advocated for use early in the course of attacks Therapeutic agents available for the treatment of acute attacks of HAE are summarized in Table 3[23,32-34]; see also the following discussion regarding differential availability of these agents.

**Table 3:** **Treatment summary of emergency care available in the US to patients experiencing acute attacks of hereditary angioedema**[23,32-34]

**Therapy and indication**	**Dosage**	**Monitoring tests**
**C1 esterase inhibitor** [human] (Berinert; CSL Behring)	20 U/kg body weight IV at a rate of 4 ml/ minute	· Monitor patients with known risk factors for thrombotic events
*Indicated for the treatment of acute abdominal or facial attacks of HAE in adult and adolescent patients*		
		· Epinephrine should be immediately available to treat any acute severe hypersensitivity reactions following discontinuation of administration
**Plasma kallikrein inhibitor** (Kalbitor [ecallantide]; Dyax Corp)	30 mg (3 ml) SC in three 10-mg (1 ml) injections. If attack persists, additional dose of 30 mg (3 ml) may be administered within a 24-h period	· Given the similarity in hypersensitivity symptoms and acute HAE symptoms, monitor patients closely for hypersensitivity reactions
*Indicated for attacks at all anatomic sites*		
		· Administer in a setting equipped to manage anaphylaxis and HAE
**Fresh-frozen plasma**	2 U at 1 to 12 h before the event (only for use when C1-INH concentrate is not available)	· Baseline: liver function tests, hepatitis virology
**Bradykinin** β2 **receptor antagonist**	30 mg (3 ml) injected SC in the abdominal area. If attack persists, additional injections of 30 mg (3 ml) may be administered at intervals of ≥6 h. No more than 3 injections in 24 hours	For patients who never received Firazyr previously, the first treatment should be given in a medical institution or under the guidance of a physician
(Firazyr [icatibant]; Shire Orphan Therapies)		
*Indicated for attacks at all anatomic sites*		

*C1-INH* C1 esterase inhibitor, *IV* intravenously, *SC* subcutaneously.

### Drug therapy for hereditary angioedema

Before 2008, no drug had been approved in the US that was predictably effective for the treatment of acute attacks of HAE [35]. Until recently, the mainstay of emergency medical treatment has been IV fresh frozen plasma (FFP) and epsilon-aminocaproic acid [36]. Both anecdotal and published reports suggest that FFP replaces plasma C1-INH, thereby aborting an ongoing attack. There is, however, a theoretical and demonstrated increased risk of worsened swelling following administration of FFP during an acute attack, which is most likely due to the concurrent replacement of both plasma proteases and substrates that are involved in the mediation of an attack. Only anecdotal reports suggest that epsilon-aminocaproic acid offers minimal relief during an acute attack of HAE; however, there is no published evidence that it provides significant benefit [5,35].

#### C1-INH replacement therapy

C1-INH replacement therapy functions to restore the missing C1-INH in patients with HAE. Berinert is a human, plasma-derived, pasteurized form of C1-INH that was approved by the US Food and Drug Administration (FDA) in 2009 for the treatment of acute abdominal, facial, and, more recently, laryngeal attacks of HAE in adult and adolescent patients [32]. C1-INH concentrate has been available in Europe for more than 20 years and is considered the standard of care for the treatment of HAE in many countries. Pasteurized and nanofiltered C1-INH is provided as a single-use vial that contains 500 units of C1 esterase inhibitor as a lyophilized concentrate. Each vial must be reconstituted with 10 mL of diluent (sterile water) provided. C1-INH concentrate must be administered using aseptic technique at a dose of 20 units per kilogram of body weight by IV injection (Table 3) [32,37]. Recently, the FDA has approved self-administration of Berinert by patients.

#### Plasma kallikrein inhibitor

In 2009, the FDA granted approval to ecallantide (Kalbitor), for the treatment of acute attacks of HAE in patients 16 years of age and older [33]. However, the European Union (EU) recently rendered a negative opinion regarding its approval. Ecallantide is a plasma kallikrein inhibitor that is effective against attacks of HAE at any anatomic location, including abdominal/gastrointestinal, laryngeal, and peripheral attacks (Table 3). Ecallantide binds to plasma kallikrein and blocks its binding site, inhibiting the conversion of HMWK to bradykinin. By directly inhibiting plasma kallikrein, ecallantide reduces the conversion of HMWK to bradykinin and thereby treats symptoms that occur during acute episodic attacks of HAE.

Two randomized placebo-controlled trials demonstrated that a 30 mg subcutaneous dose of ecallantide significantly reduced the duration of symptoms in patients with HAE [38,39]. The most commonly reported adverse events were headache (8%), nausea (5%), and diarrhea (4%) [38]. Because of the 2.9% incidence of anaphylaxis observed in clinical trials, the FDA has given ecallantide a black box warning, which requires the drug to be administered by a trained healthcare professional with emergency therapy readily available to treat an allergic reaction should one occur [33].

#### Bradykinin receptor antagonist

The bradykinin receptor blocker icatibant (Firazyr) (30 mg injected subcutaneously) is a synthetic, 10 amino acid, short-acting, and highly selective competitive bradykinin β2 receptor antagonist [34,38,40]. Three trials have examined the safety and efficacy of icatibant in HAE [41,42]. These studies showed a decrease in median time to clinically significant symptom relief. This decrease was statistically significant in the For Angioedema Subcutaneous Treatment (FAST)-2 and FAST-3 trials [41,42]. Adverse reactions consisted of injection site reactions in more than 90% of subjects, pyrexia, and elevated transaminase levels [41,42]. No anaphylaxis was reported. Since bradykinin is thought to play a major role in the antihypertensive effect of ACE inhibitors, the icatibant package insert reports that any bradykinin β2 receptor antagonist has the potential to attenuate the antihypertensive effect of ACEIs [34].

#### Treatment variations by region and country

Because certain treatment options may be licensed in some countries but not in others, the treatment of HAE differs across countries. Phase III clinical trials are ongoing in the US for specific agents, and the standard of care for the treatment of HAE will continue to evolve as data from these trials become available. Rigorous phase IV clinical trials will further delineate the long-term safety and efficacy of the differing treatments. Data from all trials will be used to update international and national HAE databases and registries [43].

Icatibant is approved for the treatment of acute attacks of HAE in the EU and the US. The recent FDA indication for icatibant allows patients with HAE aged 18 years or older to self-administer the medication [8,43].

Another difference across countries regarding the treatment of HAE is the use of a recombinant C1-INH (conestat alfa, Rhucin), which is produced in transgenic rabbit milk. Recombinant C1-INH is currently under FDA review; in June 2010, the Committee for Medicinal Products for Human Use of the European Medicines Agency delivered a positive opinion on the use of recombinant C1-INH for the treatment of acute attacks in patients with HAE [43].

Regardless of the agent selected for acute attacks of HAE, the patient and/or healthcare provider needs to be able to differentiate the progression of an HAE attack from that of an allergic reaction, as there are many similar features, so that erroneous treatments are not provided and erroneous diagnoses are not made. Furthermore, patients trained to self-administer these agents should be advised that if they are experiencing facial, neck, and/or throat swelling, they should go to the closest emergency department for observation after taking their HAE medication.

## Conclusions

The advent of innovative pharmacologic treatment options for acute angioedema, catalyzed by an improved understanding of pathophysiologic processes, has made possible disease-specific therapies that have a positive impact on morbidity and mortality. The ability of the emergency department physician to rapidly differentiate between the various forms of angioedema is paramount to the successful implementation of appropriate treatment for these patients.

## Abbreviations

AAE: Acquired angioedema; ACEI: Angiotensin-converting enzyme inhibitor; APP: Aminopeptidase P; ARB: Angiotensin receptor blocker; C1-INH: C1 esterase inhibitor; FFP: Fresh frozen plasma; HAE: Hereditary angioedema; HMWK: High-molecular-weight kininogen; IAE: Idiopathic angioedema; MHC2: Major histocompatibility complex 2; MI: Myocardial infarction; NSAID: Non-steroidal anti-inflammatory drug; PAE: Pseudoallergic angioedema.

## Competing interests

The manuscript was financially supported by Dyax Corp. (Cambridge, MA).

## Authors’ contributions

JAB and JM meet the criteria for authorship as recommended by the International Committee of Medical Journal Editors (ICMJE), were fully responsible for all content and editorial decisions, retained full control over all content contained in this manuscript, and were involved with all stages of manuscript development. They received no honorarium for their roles as authors of this manuscript. Editorial and writing assistance in the development of this manuscript in the form of drafting and revising content based on specific direction from the authors, collation of author comments, editing, referencing, manuscript formatting, and creation of figures was provided by Publication CONNEXION (Newtown, PA). All authors read and approved the final manuscript.

The manuscript was financially supported by Dyax Corp. (Cambridge, MA). The Medical Affairs department at Dyax Corp. was allowed several courtesy scientific accuracy reviews by the authors and provided feedback to the authors for their consideration. Dyax Corp. was not involved in the writing or editing of this manuscript and was not permitted to censor any content from the authors.
